# Development of a monoclonal antibody to study MARCH6, an E3 ligase that regulates proteins that control lipid homeostasis

**DOI:** 10.1016/j.jlr.2024.100650

**Published:** 2024-09-19

**Authors:** Shimeng Xu, Linda Donnelly, Daniel L. Kober, Myra Mak, Arun Radhakrishnan

**Affiliations:** 1 Department of Molecular Genetics, University of Texas Southwestern Medical Center, Dallas, TX; 2 Department of Biochemistry, University of Texas Southwestern Medical Center, Dallas, TX

**Keywords:** antibodies, cholesterol/biosynthesis, lipid droplets, sterols

## Abstract

Membrane-associated ring-CH-type finger 6 (MARCH6), also designated as TEB4 or RNF176, is an E3 ligase that is embedded in membranes of the endoplasmic reticulum where it ubiquitinates many substrate proteins to consign them to proteasome-mediated degradation. In recent years, MARCH6 has been identified as a key regulator of several metabolic pathways, including cholesterol and lipid droplet homeostasis, protein quality control, ferroptosis, and tumorigenesis. Despite its importance, there are currently no specific antibodies to detect and monitor MARCH6 levels in cultured cells and animals. Here, we address this deficiency by generating a monoclonal antibody that specifically detects MARCH6 in cultured cells of insect, mouse, hamster, and human origin, as well as in mouse tissues, with minimal cross-reactivity against other proteins. We then used this antibody to assess two properties of MARCH6. First, analysis of mouse tissues with this antibody revealed that the liver contained the highest levels of March6. Second, analysis of five different cell lines with this antibody showed that endogenous levels of MARCH6 are unchanged as the cellular content of cholesterol is varied. This reagent promises to be a useful tool in interrogating additional signaling roles of MARCH6.

Membrane-associated ring-CH-type finger 6 (MARCH6), also designated as TEB4 or RNF176, is an E3 ligase that is embedded in membranes of the endoplasmic reticulum (ER) (1). The role of E3 ligases is to ubiquitinate substrate proteins to target them for degradation by the ubiquitin-proteasome pathway (2). By triggering the degradation of substrate proteins, E3 ligases control the levels of many key enzymes and play a crucial role in regulating diverse cellular signaling pathways (3).

The substrates of MARCH6 are varied, ranging from squalene monooxygenase (SQLE), a key enzyme in the cholesterol biosynthetic pathway (4, 5), to other proteins involved in cholesterol and lipid droplet homeostasis (6, 7, 8, 9), protein quality control (10, 11, 12, 13), ferroptosis (14, 15), and tumorigenesis (16, 17). Despite its importance in regulating many metabolic pathways, current methods to detect and monitor MARCH6 levels in cultured cells and animals are limited (8, 10, 18). The most widely used antibody for MARCH6 for Western blot analysis displays weak specific signal with substantial background that obscures any meaningful analysis of this protein (14, 19, 20, 21).

In this report, we addressed this deficiency by generating a monoclonal antibody, designated as IgG-26F12c, using purified full-length human MARCH6 as an immunogen. Unlike existing antibodies for MARCH6, IgG-26F12c specifically detects MARCH6 with minimal cross-reactivity for other proteins. IgG-26F12c efficiently detects MARCH6 in mouse tissues as well as in cultured cells of insect, mouse, hamster, and human origin, finally allowing detailed studies of MARCH6 in various cellular contexts.

## Materials and Methods

### Experimental models

#### Animals

Two strains of mice were used in these studies: i) 6- to 8-week-old male NZBWF1/J mice (Jackson Laboratories, Cat# 100008) were used for immunization studies and ii) 8-week-old male C57BL/6J mice (Jackson Laboratories, Cat# 000664) were used for assessing the tissue distribution of March6. Both strains of mice were housed at the University of Texas Southwestern Medical Center Animal Resource Center in 12 h light/12 h dark cycles (9 am–9 pm as light cycle) and given ad libitum access to water and food (Harlan Teklad Rodent Diet 2018 as chow diet). All experiments were conducted with the approval and oversight of University of Texas Southwestern Medical Center’s Institutional Animal Care & Use Committee (IACUC; APN: 2017-102391; 2015-101135).

#### Cell lines

3T3-L1 cells (mouse embryo fibroblast; ATCC, Cat# CL-173) were maintained in medium A. A549 cells (human lung epithelial; ATCC, Cat# CCL-185) were maintained in medium B. CHO-K1 cells (Chinese hamster ovary; ATCC, Cat# CCL-61) were maintained in medium B. HEK293A cells (human embryonic kidney; Ref. (22)) were maintained in medium C. HEK293S GnTI^-^ cells (human embryonic kidney; ATCC, Cat# CRL-3022) were maintained in medium D. HEK293T (Lenti-X) cells (human embryonic kidney; Clontech, Cat# 632180) were maintained in medium C. HeLa cells (human uterus epithelial; ATCC, Cat# CRL-2) were maintained in medium E. HuH7 cells (human liver epithelial; Ref. (23)) were maintained in medium C. Sf9 cells (*Spodoptera frugiperda*; Invitrogen, Cat# 11496015) were maintained in medium F. SV589 cells (human skin fibroblast; Ref. (24)) were maintained in medium G. 3T3-L1, A549, CHO-K1, HEK293A, and HeLa cells were grown in monolayer in an incubator maintained at 37°C with atmosphere of 8.8% CO_2_. HEK293T (Lenti-X), HuH7, and SV589 cells were grown in monolayer in an incubator maintained at 37°C with atmosphere of 5% CO_2_. HEK293S GnTI^-^ cells were grown in suspension in baffled flasks as previously described (25) on an orbital shaking platform (130 rpm) in an incubator maintained at 37°C, 8% CO_2_, and 70% humidity. Sf9 cells were grown in suspension on an orbital shaking platform (120 rpm) in an incubator maintained at 27°C and ambient atmospheric conditions. To guard against potential genomic instability, all cell lines were passaged for less than 6 weeks, after which a fresh aliquot of cells was thawed and propagated. All cell lines were confirmed to be free of mycoplasma contamination using the LookOut Mycoplasma PCR Detection Kit (Sigma, Cat# MP0035).

### Experimental reagents

#### Culture media

Medium A is DMEM (low glucose) (Sigma, Cat# D6046) supplemented with 10% (v/v) newborn calf serum (Sigma, Cat# N4637), 100 units/ml penicillin (Sigma, Cat# P0781), and 100 μg/ml streptomycin sulfate (Sigma, Cat# P0781). Medium B is a 1:1 mixture of DMEM and Ham’s F-12 medium (Corning, Cat# 10-090-CV) supplemented with 5% (v/v) FCS (Sigma, Cat# F2442), 100 units/ml penicillin, and 100 μg/ml streptomycin sulfate. Medium C is DMEM (high glucose) (Sigma, Cat# D6429) supplemented with 5% (v/v) FCS, 100 units/ml penicillin, and 100 μg/ml streptomycin sulfate. Medium D is FreeStyle 293 medium (Thermo Fisher Scientific, Cat# 12338018) supplemented with 2% (v/v) FCS, 100 units/ml penicillin, and 100 μg/ml streptomycin sulfate. Medium E is MEM (Corning, Cat# 10-010-CV) supplemented with 5% (v/v) FCS, 1x nonessential amino acids (Corning, Cat# 25-025-CI), 1 mM sodium pyruvate (Corning, Cat# 25-000-CI), 100 units/ml penicillin, and 100 μg/ml streptomycin sulfate. Medium F is SF900 II serum free medium (GIBCO, Cat# 10902104). Medium G is DMEM (low glucose) supplemented with 5% (v/v) FCS, 100 units/ml penicillin, and 100 μg/ml streptomycin sulfate. Medium H is DMEM (high glucose) supplemented with 5% (v/v) newborn calf lipoprotein-deficient serum (LPDS) (26), 50 μM sodium compactin (27), 50 μM sodium mevalonate (27), 100 units/ml penicillin, and 100 μg/ml streptomycin sulfate. Medium I is DMEM (low glucose) supplemented with 5% (v/v) LPDS, 50 μM sodium compactin, 50 μM sodium mevalonate, 100 units/ml penicillin, and 100 μg/ml streptomycin sulfate. Medium J is DMEM (high glucose) supplemented with 10% (v/v) FCS, 100 units/ml penicillin, and 100 μg/ml streptomycin sulfate.

#### Buffers

Buffer A contains 50 mM Hepes-NaOH (pH 7.5), 500 mM NaCl, 0.5 mM tris(2-carboxyethyl)phosphine (TCEP), 20% (v/v) glycerol, 1% (w/v) lauryl maltose neopentyl glycol, and 0.1% (w/v) cholesteryl hemisuccinate (CHS). Buffer B contains 50 mM Hepes-NaOH (pH 7.5), 150 mM NaCl, 0.5 mM TCEP, 10% (v/v) glycerol, 0.1% (w/v) glyco-diosgenin, and 0.01% (w/v) CHS. Buffer C contains 50 mM Hepes-NaOH (pH 7.5), 150 mM NaCl, 0.5 mM TCEP, 0.02% (w/v) glyco-diosgenin, and 0.002% (w/v) CHS. Buffer D contains 50 mM Tris-HCl (pH 7.4), 150 mM NaCl, 1% (w/v) SDS, 1 mM EDTA, and 1 mM EGTA. Buffer E contains 250 mM Tris-HCl (pH 6.8), 10% (w/v) SDS, 0.2% (w/v) bromophenol blue, 25% (v/v) glycerol, and 5% (v/v) β-mercaptoethanol. Buffer F contains 50 mM Tris-HCl at pH 7.4, 150 mM NaCl, and 1% (v/v) Triton X-100. A protease inhibitor stock solution was prepared by dissolving two protease inhibitor tablets (Thermo Fisher Scientific, Cat# A32965) in 1 ml H_2_O.

#### Expression plasmids

p3xFLAG-MARCH6(C9A) is a plasmid in the pEZT expression vector that encodes, in sequential order from the NH_2_ terminus, a 31-aa linker that includes three copies of the FLAG epitope (MDYKDDDDKGSDYKDDDDKGSDYKDDDDKTG), followed by full-length human MARCH6 (aa 1–910) containing one point mutation (C9A). The coding sequence for MARCH6 was generated as a codon-optimized dsDNA block (IDT Technologies) and subcloned into the pEZT vector using Gibson assembly methods (NEB). Using p3xFLAG-MARCH6(C9A) as template, we generated a new plasmid, p3xFLAG-MARCH6-1D4, with two modifications: i) the point mutation (C9A) was reverted back to the WT cysteine (C9) and ii) a 1D4 epitope (TETSQVAPA) was appended to the COOH terminus.

#### Generation of cell lines lacking MARCH6

We used the Benchling CRISPR guide RNA design tool (https://www.benchling.com/crispr) to design guide RNAs targeting either exon 15 (5ʹ-TCTCAGTAGTAGAATGAAGG-3′, designated as KO1) or exon 4 (5ʹ-TTGGTTTCATTATACACTTG-3′, designated as KO2) of MARCH6. The guide RNAs were then cloned into the lentiviral vector lentiCRISPRv2 (Addgene, Cat# 98290). For lentivirus production, Lenti-X cells were set up on day 0 in medium C at a density of 2 × 10^6^ cells/10 cm dish. On day 1, cells were transfected with three plasmids: i) 5 μg puromycin-selectable lentiCRISPRv2 plasmid encoding a guide targeting either exon 15 or exon 4 of MARCH6 as described above; ii) 3 μg psPAX2 plasmid encoding HIV gag polymerase (Addgene, Cat# 12260); and iii) 2 μg pMD2.G plasmid encoding vesicular stomatitis virus glycoprotein (Addgene, Cat# 12259). Transfections were carried out using X-tremeGENE HP (Sigma, Cat# XTGHP-RO) according to the manufacturer’s protocol. On day 2, 24 h after transfection, media was removed and replaced with medium C supplemented with additional FCS (final concentration: 30% (v/v)) and 2 mM glutamine. After 48 h, the media was removed, filtered through a 0.45 μm PVDF filter, and either used immediately or stored at −80°C for future use. Using these lentiviruses, we generated versions of two cultured cell lines (HuH7 and SV589) that were deficient in MARCH6. To generate HuH7 cells that lacked MARCH6, HuH7 cells were set up on day 0 in medium C in 100 mm dishes at a density of 200,000 cells per dish. On day 1, the media was removed and replaced with 7 ml of fresh medium C plus 1 ml of the lentiviral medium prepared above. Each dish was supplemented with 8 μg/ml polybrene, a cationic polymer that increases the efficiency of lentiviral transduction. After 24 h, the media was removed and replaced with fresh medium C supplemented with 3 μg/ml puromycin. The transduced cells were then selected by virtue of their ability to survive in the presence of puromycin for 14 days. During this 14-day selection period, media was removed every 2–3 days and replaced with fresh medium C supplemented with 3 μg/ml puromycin. The surviving colonies were picked, expanded, and screened by immunoblotting for MARCH6. The clones lacking MARCH6 immunoblot signal were further subjected to limiting dilution to yield single clone KO cell lines. The MARCH6 genes in these lines were analyzed by genomic sequencing using primers to amplify exon 15 (KO1; forward primer 5′-tttgttagtggtggtagaaattggagtattcc-3′ and reverse primer 5′-gcatcactgtagagcatgacattgtatgg-3′) and exon 4 (KO2; forward primer 5′-atgtactggcagtattaagtttatccatcaaga-3′ and reverse primer 5′-gcagcaacattttctgcaccattt-3′). Sequencing analysis revealed one cell line that had a 1 bp deletion in both alleles of exon 15 resulting in a premature stop codon (Fig. 2A), which is hereafter designated as HuH7 (MARCH6-KO1). Sequencing analysis of the cell line derived from targeting exon 4 revealed a 144 bp deletion in both alleles (Fig. 2A), and this cell line is hereafter designated as HuH7 (MARCH6-KO2). We also generated MARCH6-deficient SV589 cells using the same method described above with two exceptions: i) medium G was used instead of medium C and ii) selection was carried out with 1.5 μg/ml puromycin. Using the same primers as listed above, we found that the cell line derived from targeting exon 15 had a 4 bp deletion in both alleles of this exon resulting in a premature stop codon (Fig. 7A), and this cell line is hereafter designated as SV589 (MARCH6-KO1). The cell line derived from targeting exon 4 had a 144 bp deletion in one allele (resulting in a large truncation) and a 44 bp deletion in the other allele (resulting in a premature stop codon) (Fig. 7A), and this cell line is designated as SV589 (MARCH6-KO2).

**Fig. 1:**
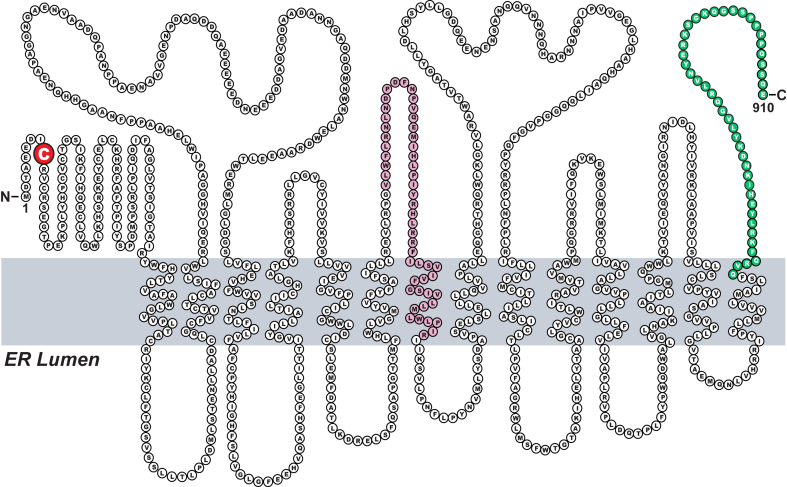
Topology model of the human ortholog of MARCH6. MARCH6 is embedded in the ER membrane through 14 transmembrane helices. The NH_2_ terminus contains a RING domain (aa 9–55) that faces the cytosol and carries out the protein’s E3 ligase activity, which is abolished by mutation of a key cysteine residue (C9, *red*). Peptides corresponding to residues 450–500 (*pink*) or COOH-terminal residues 866–910 (*green*) were used as immunogens to generate MARCH6 antibodies by Bethyl Laboratories (Cat#A304-174) and Thermo Fisher Scientific (Cat# PA5-103816), respectively. ER, endoplasmic reticulum; MARCH6, membrane-associated ring-CH-type finger 6.

**Fig. 2:**
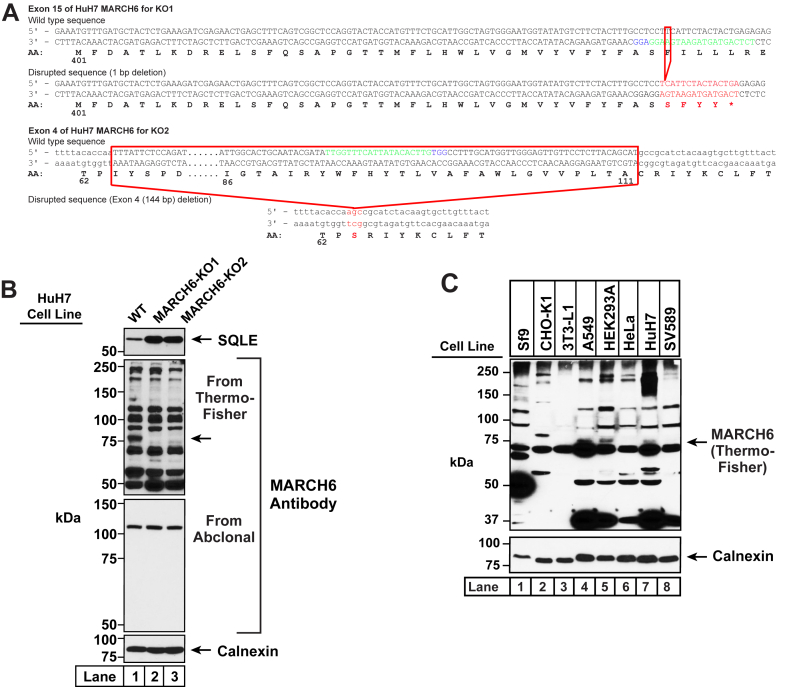
Detection of MARCH6 in HuH7 cells. A: Strategy for generating MARCH6-deficient HuH7 cells using CRISPR-Cas9 technology. Two guide RNAs were designed to target and disrupt either exon 15 or exon 4 of human MARCH6 (also designated as *MARCHF6*). The 20-nucleotide target sequence is shown in green and the NGG PAM sequence is in blue. Genomic sequencing of cells where exon 15 was disrupted revealed a 1-bp deletion (*red box*) that resulted in a truncated transcript encoding amino acids 1–437 of MARCH6 followed by four residues (*red*) and a stop codon (∗), and this cell line is designated as MARCH6-KO1. Genomic sequencing of cells where exon 4 was disrupted revealed a deletion of the whole exon 4 that resulted in deletion of 49 residues and insertion of a serine (*red*), and this cell line is designated as MARCH6-KO2. B: Immunoblot analysis of endogenous MARCH6. On day 0, HuH7 cells (WT, MARCH6-KO1, and MARCH6-KO2) were set up in medium C at a density of 4 × 10^5^ cells per well of a 6-well plate. On day 1, the medium was removed, cells were washed twice with 500 μl of PBS, harvested, and equal aliquots of cell lysates were subjected to immunoblot analysis with two commercially available MARCH6 antibodies as described in Methods. C: Analysis of endogenous MARCH6 in different cell lines using a commercially available antibody. Sf9 cells were set up on day 0 in medium F at a density of 1 × 10^6^ cells/ml (total volume of 25 ml) in a 125 ml flask and incubated at 27°C while shaking at 120 rpm. On day 1, 1 ml of Sf9 cells were harvested and processed for immunoblot analysis as described in Methods (*lane 1* in all panels). All other cell lines were set up on day 0 at a density of 4 × 10^5^ cells per well of a 6-well plate (SV589 cells, medium G; HuH7 and HEK293A cells, medium C; CHO-K1 and A549 cells, medium B; HeLa cells, medium E; and 3T3-L1 cells, medium A). On day 1, the medium was removed, cells were washed twice with 500 μl of PBS, harvested, and equal aliquots of cell lysates were subjected to immunoblot analysis as described in Methods (*lanes 2–8* in all panels). MARCH6, membrane-associated ring-CH-type finger 6.

**Fig. 3:**
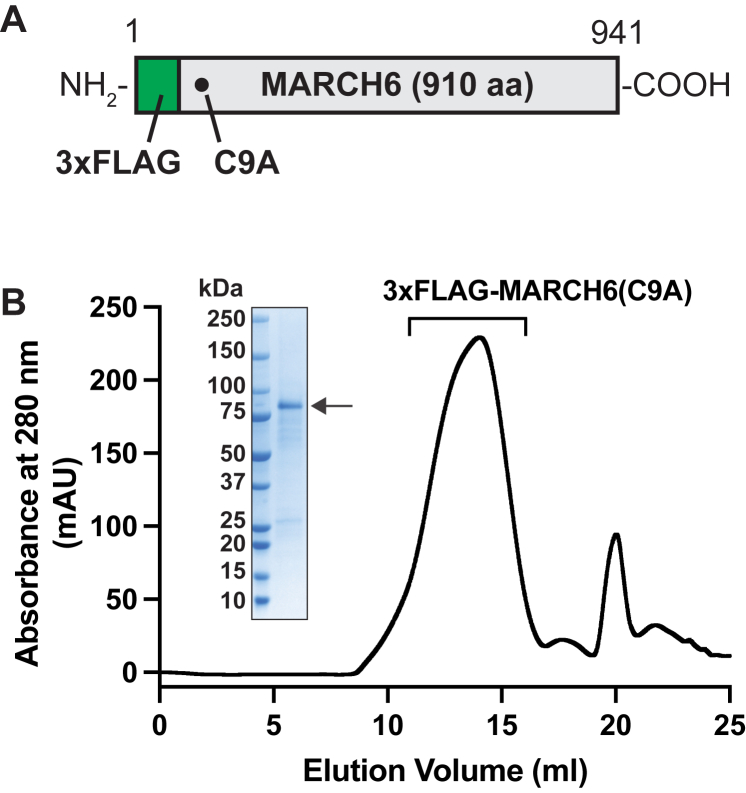
Purification of recombinant human MARCH6(C9A). A: Schematic of p3xFLAG-MARCH6(C9A) plasmid. This plasmid encodes full-length human MARCH6 (aa 1–910) containing three copies of the FLAG epitope at its NH_2_ terminus (*green*) and one point mutation (C9A) that disrupts its catalytic activity. B: Gel filtration of purified 3xFLAG-MARCH6(C9A). Recombinant 3×FLAG-MARCH6(C9A) was purified in GDN detergent micelles as described in Methods and subjected to gel filtration chromatography on a Superose 6 Increase column. Peak fractions were pooled, and an aliquot was subjected to SDS/PAGE followed by Coomassie staining (*inset*). GDN, glyco-diosgenin; MARCH6, membrane-associated ring-CH-type finger 6.

**Fig. 4:**
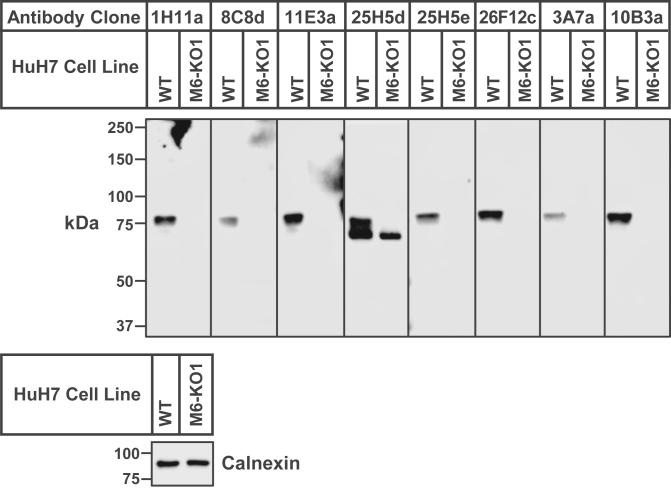
Primary screen of MARCH6 antibodies. Antibodies against human MARCH6(C9A) were generated as described in Methods. Initial ELISA screening identified 219 positive clones that were then analyzed by immunoblot analysis. On day 0, WT and MARCH6-KO1 (M6-KO1) HuH7 cells (see Fig. 2) were set up in medium C at a density of 4 × 10^5^ cells per well of a 6-well plate. On day 1, the medium was removed, cells were washed twice with 500 μl of PBS, harvested, and equal aliquots of cell lysates were subjected to immunoblot analysis with either the conditioned media from each of the 219 ELISA-positive clones or with an antibody against calnexin (*loading control*). Results are shown for 8 of the 219 samples, which all detect an ∼75-kDa band in WT, but not M6-KO1 cells. MARCH6, membrane-associated ring-CH-type finger 6.

**Fig. 5:**
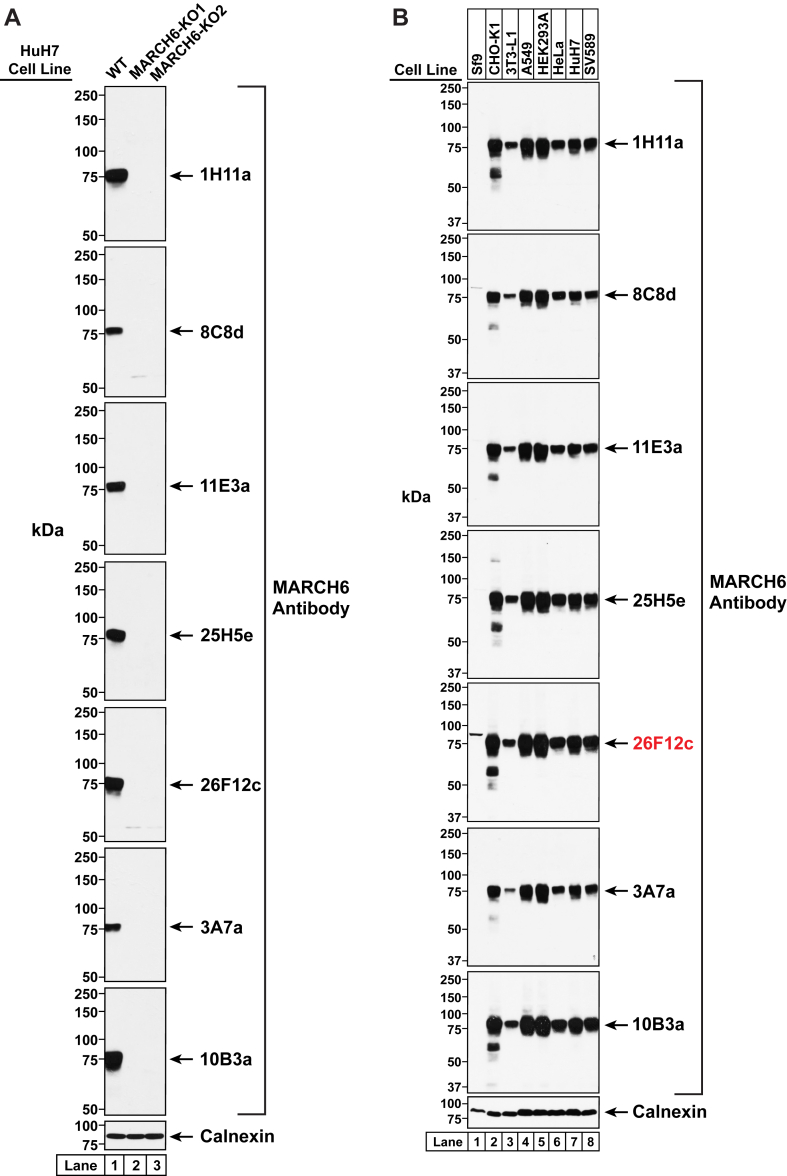
Analysis of purified MARCH6 antibodies. The 7 ELISA-positive clones that specifically detected MARCH6 in Fig. 4 were further subcloned by rapid limiting dilution, and antibodies were purified as described in Methods. These seven purified antibodies were analyzed for specificity and applicability to diverse cell lines. A: Specificity of MARCH6 antibodies. On day 0, HuH7 cells (WT, MARCH6-KO1, and MARCH6-KO2) were set up in medium C at a density of 4 × 10^5^ cells per well of a 6-well plate. On day 1, the medium was removed, cells were washed twice with 500 μl of PBS, and harvested. Equal aliquots of cell lysates were subjected to immunoblot analysis, as described in Methods, with either each of the seven purified antibodies or anti-calnexin (*loading control*). All MARCH6 antibodies were tested at concentrations of 1 μg/ml. B: Analysis of MARCH6 with purified antibodies in different cell lines. Sf9 cells were set up on day 0 in medium F at a density of 1 × 10^6^ cells/ml (total volume of 25 ml) in a 125 ml flask and incubated at 27°C while shaking at 120 rpm. On day 1, 1 ml of Sf9 cells were harvested and processed for immunoblot analysis as described in Methods (*lane 1* in all panels). All other cell lines were set up on day 0 at a density of 4 × 10^5^ cells per well of a 6-well plate (SV589 cells, medium G; HuH7 and HEK293A cells, medium C; CHO-K1 and A549 cells, medium B; HeLa cells, medium E; and 3T3-L1 cells, medium A). On day 1, the medium was removed, cells were washed twice with 500 μl of PBS, harvested, and equal aliquots of cell lysates were subjected to immunoblot analysis as described in Methods (*lanes 2–8* in all panels). All MARCH6 antibodies were tested at concentrations of 1 μg/ml. IgG-26F12c detected MARCH6 with high specificity in all cell lines tested and was chosen for use in subsequent experiments (*highlighted in red*). MARCH6, membrane-associated ring-CH-type finger 6.

**Fig. 6:**
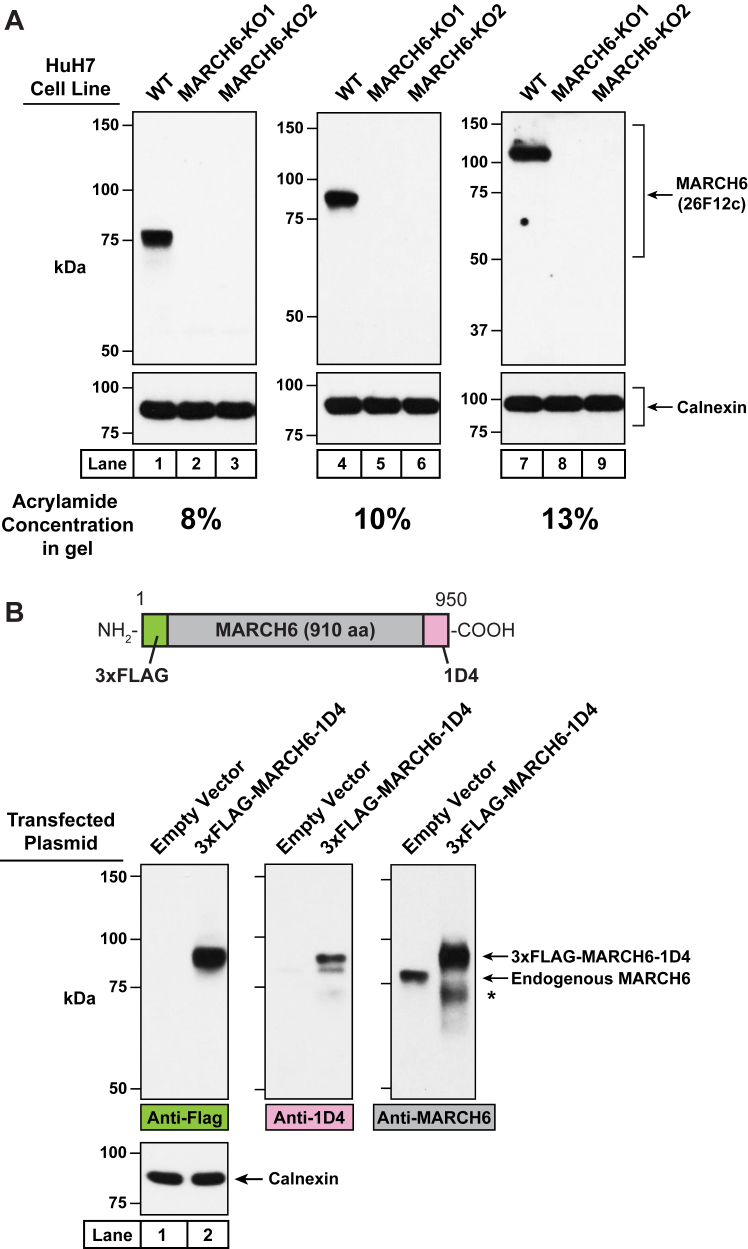
Comparison of the electrophoretic migration of MARCH6 on gels with different acrylamide concentrations and analysis of the stability of epitope tags at the NH_2_ and COOH termini of MARCH6. A: On day 0, HuH7 cells (WT, MARCH6-KO1, and MARCH6-KO2) were set up in medium C at a density of 4 × 10^5^ cells per well of a 6-well plate. On day 1, the medium was removed, cells were washed twice with 500 μl of PBS, and harvested. Equal aliquots of the same cell lysates were subjected to SDS-PAGE on three different gels containing the indicated concentrations of acrylamide, followed by immunoblot analysis with either IgG-26F12c (1 μg/ml) or anti-calnexin (*loading control*). B: On day 0, HEK293T cells were set up in medium C supplemented with 2 mM L-glutamine at a density of 4 × 10^5^ cells per well of a 6-well plate. On day 1, the medium was removed, and fresh media (same as above) was added to each well, and cells were transfected with 2 μg of either empty pEZT vector or p3xFLAG-MARCH6-1D4 using X-tremeGENE HP as the transfection reagent according to the manufacturer’s instructions. On day 2, the medium was removed, cells were washed twice with 500 μl of PBS, and harvested. Equal aliquots of cell lysates were subjected to 8% SDS-PAGE and immunoblot analysis with either anti-FLAG, anti-1D4, IgG-26F12c, or anti-calnexin antibodies. (∗, band of unknown origin possibly related to degradation of overexpressed 3xFLAG-MARCH6-1D4). MARCH6, membrane-associated ring-CH-type finger 6.

**Fig. 7:**
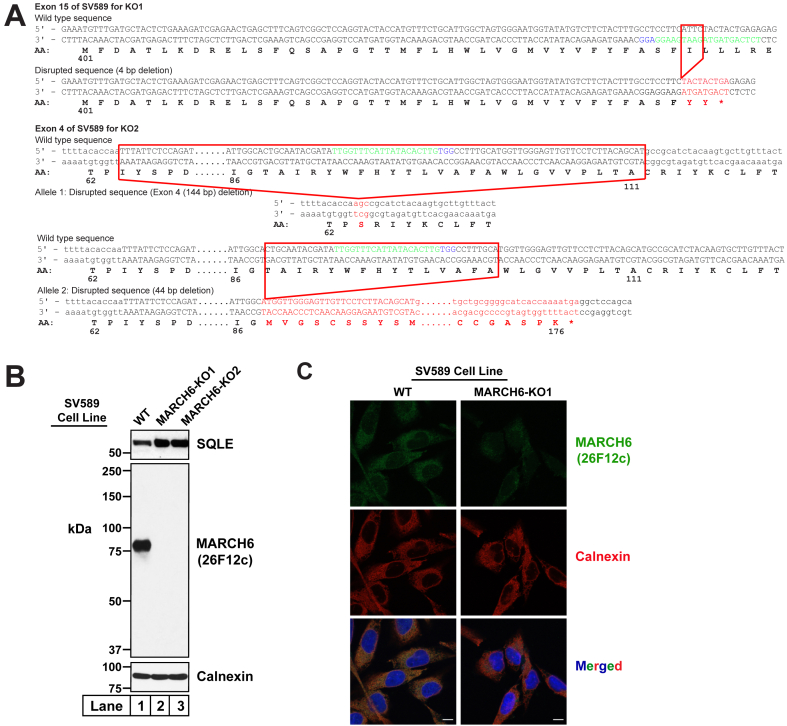
Specificity of IgG-26F12c (anti-MARCH6) in SV589 cells. A: Strategy for generating MARCH6-deficient SV589 cells using CRISPR-Cas9 technology. Two guide RNAs were designed to target and disrupt either exon 15 or exon 4 of human MARCH6 (also designated as *MARCHF6*). The 20-nucleotide target sequence is shown in green and the NGG PAM sequence is in blue. Genomic sequencing of cells where exon 15 was disrupted revealed a 4-bp deletion (*red box*) that resulted in a truncated transcript encoding amino acids 1–438 of MARCH6 followed by two residues (*red*) and a stop codon (∗), and this cell line is designated as MARCH6-KO1. Genomic sequencing of cells where exon 4 was targeted revealed that the disruption in one allele was a deletion of the whole exon 4, resulting in deletion of 49 residues and insertion of a serine (*red*), and the disruption in the other allele was a 44-bp deletion in exon 4 (*red box*) that resulted in a truncated transcript encoding amino acids 1–87 of MARCH6, followed by 89 unrelated residues (*red*) and a stop codon (∗). This cell line is designated as MARCH6-KO2. B: Immunoblot analysis of endogenous MARCH6. On day 0, SV589 cells (WT, MARCH6-KO1, and MARCH6-KO2) were set up in medium G at a density of 4 × 10^5^ cells per well of a 6-well plate. On day 1, the medium was removed, cells were washed twice with 500 μl of PBS, harvested, and equal aliquots of cell lysates were subjected to immunoblot analysis as described in Methods. C: Immunofluorescence analysis of endogenous MARCH6. On day 0, SV589 cells (WT or MARCH6-KO1) were set up in medium G at a density of 4 ×10^4^ cells per well of a 24-well plate, each well of which contained one 12 mm coverslip. On day 2, the medium was removed, cells were washed twice with 500 μl of PBS, fixed, and processed for immunofluorescence analysis as described in Methods. Scale bar, 10 μm. MARCH6, membrane-associated ring-CH-type finger 6.

#### Overexpression of recombinant MARCH6

We generated p3xFLAG-MARCH6(C9A) BacMam baculoviruses using the Bac-to-Bac expression system (Thermo Fisher Scientific) as previously described (28). HEK293S GnTI^-^ cells were thawed in medium C supplemented with additional FCS (final concentration: 10% (v/v)) and grown in monolayer culture at 37°C in 5% CO_2_. After expanding to ten 10 cm dishes, cells were sloughed off, transferred to medium D, and grown in suspension on an orbital shaker (130 rpm) in an incubator maintained at 37°C and 8% CO_2_. 4 L cultures of HEK293S GnTI^-^ cells at a density of 2.5 million cells/ml were transduced with p3xFLAG-MARCH6(C9A) baculovirus at a multiplicity of infection of 3 virions per cell. The cultures were supplemented with 2.5 mM sodium butyrate and moved to an orbital shaker (130 rpm) in a different incubator maintained at 30°C and 8% CO_2_. After incubation for 72 h, cells were pelleted by centrifugation at 3,000*g* for 30 min at 4°C, and the pellets were stored at −80°C until purification.

#### Purification of recombinant MARCH6

Cell pellets from 4 L cultures of HEK293S GnTI^-^ cells infected with p3xFLAG-MARCH6(C9A) baculovirus were thawed, disrupted by Dounce homogenization, and then lysed by resuspension in 500 ml of hypotonic lysis buffer (50 mM Hepes-NaOH pH 7.5 and 0.5 mM TCEP) supplemented with protease inhibitors (160 mg/ml benzamidine, 1 mM PMSF, 1 mM E64, and 2.5 mg/ml leupeptin). Membranes were pelleted by centrifugation at 22,000*g* for 30 min at 4°C. The supernatant was decanted, and the membranes were resuspended by Dounce homogenization in buffer A supplemented with protease inhibitors (160 mg/ml benzamidine, 1 mM PMSF, 1 mM E64, and 2.5 mg/ml leupeptin) along with benzonase (25 mU/μl). The mixture was stirred vigorously for 1 h at 4°C, followed by centrifugation at 150,000*g* for 1 h at 4°C to pellet insoluble material. The resulting supernatant was incubated with M2 FLAG affinity gel for 3 h to allow binding of FLAG-tagged proteins. The FLAG beads were then collected by centrifugation and transferred to a gravity-flow column. The beads were washed with 15 column volumes of buffer B. Bound proteins were eluted with buffer B supplemented with 400 μg/ml FLAG peptide. Eluates were concentrated to 1 ml using Amicon centrifugal concentrators (100 kDa molecular weight cutoff [MWCO]) and subjected to gel filtration on a Superose 6 Increase column equilibrated in buffer C. Protein-rich fractions were pooled, concentrated to 1 mg/ml, flash-frozen in liquid nitrogen, and stored at −80°C.

#### Development of antibodies for MARCH6

Four male New Zealand Black NZBWF1/J mice (Jackson Laboratories) were immunized with purified recombinant human MARCH6 (described above) combined in a 1:1 ratio with adjuvant (Sigma, Cat# S6322) (50 μg primary injection, followed by two 10 μg boosts at two-week intervals). Three weeks after the final injection, blood was collected from the tail vein and the mouse with the highest antibody titer was identified by ELISA. One week later, this mouse was injected on three consecutive days with 10 μg of purified MARCH6. On the fourth day, the spleen was collected from the mouse. Hybridoma cells were generated by fusing the isolated splenic lymphocytes with SP2-mIL6 mouse myeloma cells (ATCC, Cat# CRL-2016) by electro-cell fusion (ECFG21 Nepagene Super Electro-cell Fusion generator). The fused hybridoma cells were set up in Clonacell-Hy medium C (Stemcell Technologies, Cat# 03803) supplemented with 1x hypoxanthine-aminopterin-thymidine (HAT; Gibco, Cat# 21060-017), which selects for properly fused cells. One week later, dead cells were removed with Miltenyi Dead Cell Removal Kit (Miltenyi Biotec, Cat# 130-090-101). The surviving hybridomas expressing IgG1 and IgG2ab surface antibodies were enriched using Miltenyi Mouse Memory B Cell Isolation Kit (Miltenyi Biotec, Cat# 130-095-838) and labeled with FITC-MARCH6 (generated using the purified recombinant MARCH6 and the FITC Conjugation Kit (Abcam, Cat# ab188285)). After sorting on a MACSQuant Tyto FACS Cell Sorter, the double positive IgG1/IgG2ab-APC and MARCH6-FITC hybridomas were plated in 44 96-well plates using the ClonaCell-Hy Hybridoma Kit (Stemcell Technologies, Cat# 03800). One week later, 50 μl of media from each well was screened by ELISA, yielding 219 positive clones. Each of these clones was transferred into a well of a 24-well plate supplemented with 2.5 ml of culture medium (DMEM (high glucose) supplemented with 20% (v/v) FCS, 10% (v/v) NCTC-109 (Gibco, Cat# 21340-039), 1× hypoxanthine-aminopterin-thymidine (HAT), 2 mM Glutamax, 1 mM sodium pyruvate, 1x nonessential amino acids, 1× insulin-transferrin-selenium (ITS) (Gibco, Cat# 51500-056), 50 μM β-mercaptoethanol, 100 units/ml penicillin, and 100 μg/ml streptomycin sulfate). After 3–4 days, the medium from each well was screened by Western Blot of lysates from WT and MARCH6 knockout cells, and the positives were subcloned by rapid limiting dilution to 1 cell per well of a 96-well plate and screened again by ELISA and Western Blot. After this multistep screening process, antibodies were purified from hybridoma supernatants using protein G Sepharose affinity columns (Cytiva, Cat# 17061805).

#### Immunoblot analysis

For analysis of lysates from cultured cells in 6-well plates, cells were washed twice with PBS (Corning, Cat# 21-031-CV) after indicated treatments, followed by addition of 300 μl of buffer D supplemented with protease inhibitors (1:100 dilution of protease inhibitor stock solution, 50 μM PMSF, and 25 μg/ml N-acetyl-leucinal-leucinal-norleucinal [ALLN]) to each well. After vigorous shaking for 20 min, the solubilized cell lysates were transferred to 1.7 ml tubes and their protein concentrations were measured using a BCA assay kit. The lysates were then supplemented with 75 μl of buffer E, heated for 20 min at 55°C, and saved for analysis. For analysis of MARCH6 protein levels in different cell lines, cells were harvested as described above except for insect Sf9 cells, which were harvested as follows: 1 ml of Sf9 cell suspension (∼2 × 10^6^ cells per ml) was subjected to centrifugation at 800*g* for 5 min, after which the pelleted cells were resuspended in PBS and subjected to further centrifugation at 800*g* for 5 min. The resulting cell pellet was resuspended in 300 μl of buffer D and after vigorous shaking for 20 min, the solubilized cell lysates were transferred to fresh tubes and their protein concentrations were measured using a BCA assay kit. The lysates were then supplemented with 75 μl of buffer E, heated for 20 min at 55°C, and saved for analysis. Equal protein amounts from each cell line (40 μg/lane) were subjected to 8% SDS-PAGE analysis.

For analysis of MARCH6 in mice, various mouse tissues were isolated and immediately frozen in liquid nitrogen. Aliquots from each tissue were resuspended in 1 ml of buffer F supplemented with protease inhibitors (1:100 dilution of protease inhibitor stock solution, 50 μM PMSF, and 25 μg/ml ALLN), and homogenized with ceramic beads (OMNI International, Cat# 19-628). The homogenates were subjected to centrifugation at 20,000*g* for 20 min at 4°C, and the protein concentrations of the resulting supernatants (tissue lysates) were measured using a BCA assay kit. For the white adipose tissue and brown adipose tissue lysates, trichloroacetic acid (5% (w/v) final concentration) was added to precipitate proteins. After washing twice with ice-cold acetone, the protein pellets were air-dried and resuspended in buffer F, after which their protein concentrations were measured using a BCA assay kit. An aliquot containing 600 μg of each tissue lysate was supplemented with buffer F to reach a final volume of 300 μl. This mixture was supplemented with 75 μl of buffer E, heated for 20 min at 55°C, and subjected to 8% SDS-PAGE analysis.

After SDS-PAGE, proteins were either stained with Quick Coomassie Stain (Anatrace) or transferred to nitrocellulose membranes. After incubation with blocking buffer (PBS-Tween20 supplemented with 5% (w/v) nonfat milk) either for 1 h at room temperature or overnight at 4°C, the membranes were incubated with primary antibodies either for 1 h at room temperature or overnight at 4°C. The following primary antibodies, diluted in blocking buffer, were used: anti-MARCH6 (1 μg/ml, Thermo Fisher Scientific, Cat# PA5-103816; or 1:1000 dilution, Abclonal, Cat# A16096) to detect MARCH6 in cell lines, IgG-1H11a, IgG-8C8d, IgG-11E3a, IgG-25H5d, IgG-25H5e, IgG-26F12c, IgG-3A7a, IgG-10B3a (all at a concentration of 1 μg/ml) to detect MARCH6 in cell lines and mouse tissues, anti-calnexin (1:10,000, Novus Biologicals, Cat# NB100-1974) to detect calnexin, anti-FLAG (1:500, Sigma, Cat# F1804) to detect the FLAG epitope, anti-1D4 (1:1000, Cube Biotech, Cat# 40020) to detect the 1D4 epitope, IgG-22D5 (2 μg/ml) to detect SREBP2 (29) and anti-SQLE (1:2000, Proteintech, Cat# 12544-1-AP) to detect SQLE. Bound antibodies were visualized using a 1:5,000 dilution of either donkey anti-mouse IgG (Jackson ImmunoResearch) or goat anti-rabbit IgG (Jackson ImmunoResearch), each of which was conjugated to horseradish peroxidase. Membranes were either imaged with a LI-COR Odyssey® Fc (Figs. 4 and 9) or exposed to Phoenix Blue and X-Ray film (Phoenix Research Products) at room temperature for 1–120 s (all other Figures).

**Fig. 8:**
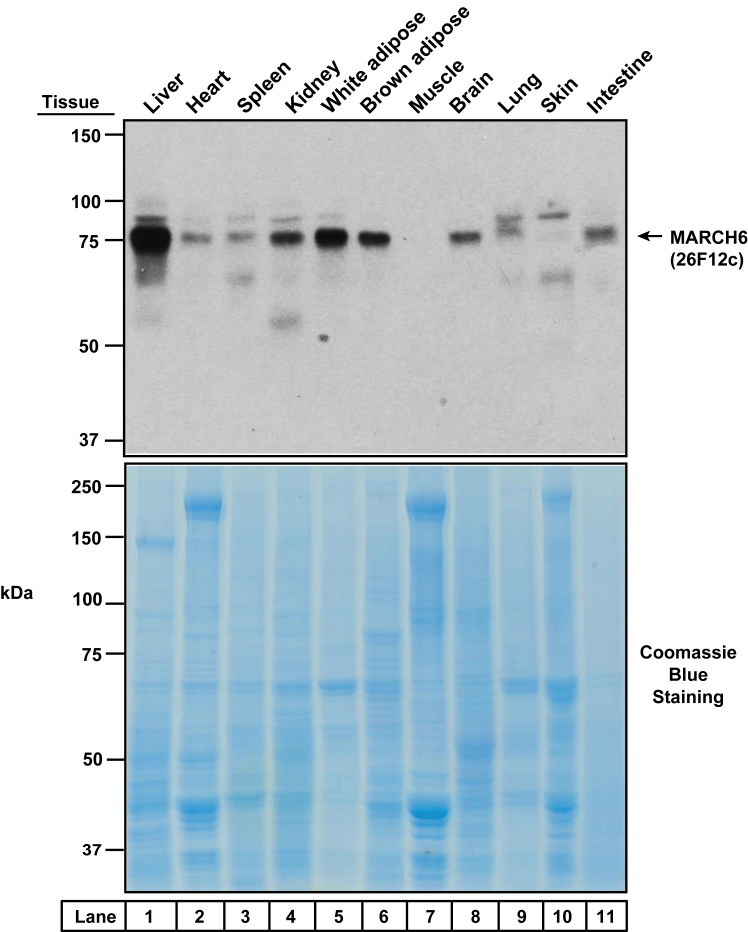
Tissue distribution of March6 in the mouse. An 8-week-old male C57BL/6J mouse was killed and the indicated 11 tissues were harvested and processed for immunoblot analysis (*top panel*) or Coomassie staining (*bottom panel*) as described in Methods. MARCH6, membrane-associated ring-CH-type finger 6.

**Fig. 9:**
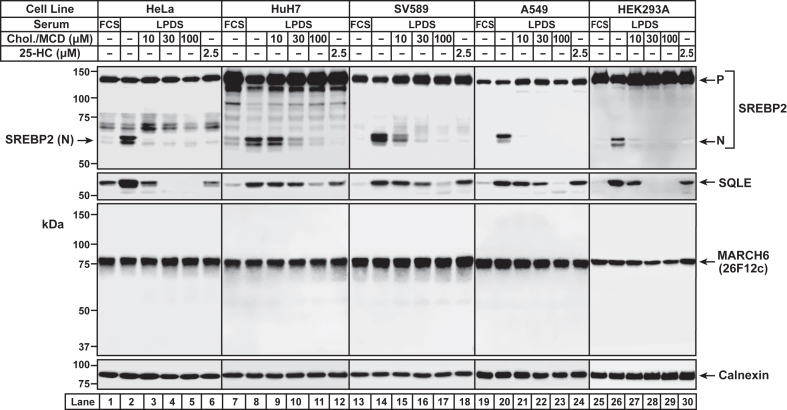
Dependence of MARCH6 levels on cellular cholesterol content in five cultured cell lines. On day 0, cells were set up at a density of 3 × 10^5^ cells per well of a 6-well plate (HeLa cells, medium E; HuH7 cells, medium C; SV589 cells, medium G; A549 cells, medium B; HEK293A cells, medium C). On day 1, the medium was removed, cells were washed twice with 2 ml of PBS, and replenished with 2 ml of media containing 5% (v/v) FCS (HeLa cells, medium C, *lane 1*; HuH7 cells, medium C, *lane 7*; SV589 cells, medium G, *lane 13*; A549 cells, medium B, *lane 19*), 10% (v/v) FCS (HEK293A cells, medium J, *lane 25*), or 5% (v/v) LPDS (HeLa cells, medium H, *lanes 2–6*; HuH7 cells, medium H, *lanes 8–12*; SV589 cells, medium I, *lanes 14–18*; A549 cells, medium H, *lanes 20–24*; and HEK293A cells, medium H, *lanes 26–30*). After incubation at 37°C for 16 h, the medium was removed, and cells were replenished with fresh aliquots of the same media as above, except that some wells were supplemented with the indicated concentrations of either cholesterol/methyl-β-cyclodextrin complexes (*lanes 3–5, 9–11, 15–17, 21–23, and 27–29*) or 25-HC (*lanes 6, 12, 18, 24, and 30*). After incubation at 37°C for 6 h, the media was removed, cells were harvested, and subjected to immunoblot analysis as described in Methods. *P*, precursor form of SREBP-2; N, cleaved nuclear form of SREBP-2; *Chol.*, cholesterol; 25-HC, 25-hydroxycholesterol; LPDS, lipoprotein-deficient serum; MARCH6, membrane-associated ring-CH-type finger 6.

#### Immunofluorescence analysis

After the indicated treatments, coverslips with SV589 cells (WT or MARCH6-KO1) were removed from the 24-well plates and placed on a strip of parafilm at room temperature. After rinsing twice with PBS, the cells were fixed with 4% (w/v) paraformaldehyde in PBS. The fixed cells were then permeabilized and blocked by treatment with 0.5% (w/v) saponin (Sigma, Cat# S4521) plus 1% (w/v) BSA (Sigma, Cat# A7030) in PBS. After treatment for 30 min, the cells were incubated with primary antibodies against calnexin and MARCH6 (IgG-26F12c) in 0.1% (w/v) saponin plus 1% (w/v) BSA in PBS for 60 min at room temperature. The cells were then incubated with Alexa Fluor 488–conjugated goat anti-mouse (Thermo Fisher Scientific, Cat# A-11001) and Alexa Fluor 594–conjugated goat anti-rabbit (Thermo Fisher Scientific, Cat# A-11037) secondary antibodies for 60 min at room temperature. Following this step, the cells were supplemented with Duolink In Situ Mounting Medium with 4',6-diamidino-2-phenylindole (DAPI) (Sigma, #DUO82040-5 ml). The slides were then sealed with nail polish and dried in the dark at room temperature before imaging. Fluorescence images were captured using a Zeiss LSM800 confocal microscope.

## Results

The human ortholog of MARCH6 is a large protein (910 aa) that resides in ER membranes and has a predicted topology as shown in Figure 1. It contains a RING domain at its NH_2_ terminus that carries out its E3 ligase activity to ubiquitinate MARCH6 substrates, and mutation of a key cysteine in this domain to alanine (C9A) abolishes its activity (Fig. 1, *highlighted in red*). The function of the rest of the protein is poorly understood. The antibody that has been used most widely to study MARCH6 was generated with a peptide immunogen comprised of amino acids 450–500 (Fig. 1, *shaded in pink*). Unfortunately, the poor specificity of this antibody has stymied studies of MARCH6, as noted in several earlier published reports (8, 18, 19, 20, 21). Moreover, this antibody (even with all its limitations) is no longer available from the vendor (Bethyl Laboratories, Cat. #A304-174). When we contacted the vendor, we were directed to an alternative antibody available from Thermo Fisher Scientific (Cat. #PA5-103816). This alternative antibody was generated with a peptide immunogen whose sequence corresponded to the COOH-terminal 45 aa of MARCH6 (Fig. 1, *shaded in green*).

To investigate MARCH6 with the commercially available antibodies, we generated cell lines that were deficient in MARCH6. We used CRISPR-Cas9 genome editing to disrupt either exon 4 or exon 15 of the gene encoding MARCH6 (*MARCHF6*) in HuH7 cells, a human liver epithelial cell line, and the resulting two cell lines are hereafter designated as MARCH6-KO1 and MARCH6-KO2 (Fig. 2A). Compared to HuH (WT) cells, the two KO cell lines had increased levels of SQLE, a known substrate of MARCH6, indicating that the KO cell lines had diminished MARCH6 activity (Fig. 2B, *top panel*). Immunoblot analysis of lysates from HuH (WT) cells with the commercially available MARCH6 antibody from Thermo Fisher Scientific revealed more than 10 distinct bands that migrated with apparent molecular masses greater than ∼50 kDa (Fig. 2B, *lane 1 of second panel*). One of these bands, migrating at ∼75 kDa and indicated by an arrow, was reduced in both KO lines (Fig. 2B, *lanes 2 and 3 of second panel*), suggesting that this band may correspond to endogenous MARCH6. We also tested the specificity of a MARCH6 antibody from a different vendor that reportedly only detects a single band in immunoblots (Abclonal, Cat# A16096). Consistent with the vendor’s assessment, immunoblot analysis of lysates from HuH (WT) cells with the Abclonal antibody revealed a single band that migrated with an apparent molecular mass greater than 100 kDa (Fig. 2B, *lane 1 of third panel*). However, the intensity of this band was unchanged in both MARCH6 knockout lines (Fig. 2B, *lanes 2 and 3 of third panel*), indicating that this band was non-specific and likely unrelated to MARCH6. We next tested whether the antibody from Thermo Fisher Scientific, which showed limited specificity for MARCH6 in HuH7 cells, could be used to assess MARCH6 in other cell lines. Immunoblot analysis of lysates from eight different cell lines of insect (Sf9), hamster (CHO-K1), mouse (3T3-L1), or human (A549, HEK293A, HeLa, HuH7, SV589) origin showed several bands of varying intensity, and it was not clear which one of these bands corresponded to MARCH6 (Fig. 2C). The band at ∼75 kDa that was reduced in HuH7 cells upon knockout of MARCH6 (see Fig. 2B, *second panel*) was largely obscured by a band at a lower molecular weight in this analysis of HuH7 cells (Fig. 2C, *lane 7*), highlighting the batch-to-batch variation of nonspecific bands detected by this antibody. Clearly, there is a need for an antibody that will allow for specific detection of MARCH6 to study its role in cellular signaling processes.

We aimed to address this need with a specific antibody against MARCH6 developed by using the entire protein (910 aa) as immunogen. To this end, we generated an expression plasmid encoding full-length human MARCH6 harboring a point mutation (C9A), with three copies of the FLAG epitope at its NH_2_ terminus (Fig. 3A). After overexpression in mammalian HEK293S GnTI^-^ cells, the resultant recombinant protein, designated as 3×FLAG-MARCH6(C9A), was purified as described in Methods. Gel filtration chromatography showed that the protein eluted as a single broad peak (Fig. 3B) and Coomassie staining (Fig. 3B, *inset*) indicated the homogeneity of purified 3xFLAG-MARCH6(C9A). This full-length, recombinant MARCH6 was injected into mice, and antibodies were generated as described in Methods. Initial ELISA screening of antibody-containing media from hybridomas set up in 96-well plates yielded 219 positive samples. We conducted immunoblot analysis of lysates from HuH7 (WT) and HuH7 (MARCH6-KO1) cells with media from each of the 219 clones, and the results for eight of these clones are shown in Figure 4. In all cases, a single band migrating with an apparent molecular mass between 75 and 100 kDa was detected in lysates of WT cells, but not of MARCH6-KO1 cells. One of these clones (25H5d) detected an additional band below the 75 kDa marker in both WT and MARCH6-KO1 cells and was excluded in subsequent analysis.

The remaining seven ELISA-positive clones were further subcloned and antibodies were purified from hybridoma supernatants as described in Methods. All 7 purified antibodies detected a single band migrating with an apparent molecular mass between 75 and 100 kDa in WT HuH7 cells (Fig. 5A, *lane 1*) and this band was absent in both of the MARCH6 KO cell lines (Fig. 5A, *lanes 2, 3*). Figure 5B shows the analysis of a diverse set of cell lines that demonstrate the ability of all seven purified antibodies to efficiently detect MARCH6 in hamster cells (CHO-K1; *lane 2*), mouse cells (3T3-L1; *lane 3*), and human cells (A549, HEK293A, HeLa, HuH7, SV589; *lanes 4–8*), with little to no cross-reactivity. We consistently detected an additional band in CHO-K1 cells that migrated between the 50 and 75 kDa markers (*lane 2*). We do not currently understand the origin of this band. One of the purified antibodies, IgG-26F12c, detected a single band likely corresponding to MARCH6 in insect cells (Sf9; *lane 1*). Due to its broad applicability and minimal cross-reactivity, IgG-26F12c was chosen as the MARCH6 antibody in subsequent experiments.

A confounding aspect of the studies so far is that the specific MARCH6 species that is detected by IgG-26F12c during 8% SDS/PAGE migrates with an apparent molecular mass of ∼75 kDa, which is lower than MARCH6’s calculated molecular weight of 102.5 kDa. The migration of purified 3xFLAG-MARCH6(C9A) during SDS/PAGE on a 4%–15% gradient gel was also at an apparent molecular mass that is lower than its calculated mass (Fig. 3B). We wondered whether the lower mass indicated posttranslational cleavage of the endogenous cellular MARCH6 or the purified, overexpressed MARCH6. However, we observed a striking effect when we subjected the same HuH7 cell lysates to SDS-PAGE on gels with different acrylamide concentrations. The band that migrated at ∼75 kDa on an 8% gel migrated at ∼85 kDa on a 10% gel and at ∼105 kDa on a 13% gel (Fig. 6A, *compare lanes 1, 4, and 7*). In all cases, no signal was observed in lysates from the two KO cell lines (Fig. 6A, *lanes 2 and 3, 5 and 6, 8, and 9*), indicating that this band was specific for MARCH6. These results suggest that the migration of MARCH6 during SDS-PAGE is sensitive to the acrylamide concentration in the gel and does not necessarily reflect the true molecular mass of the protein. To directly test the possibility of posttranslational cleavage of MARCH6, we transfected cells with an expression plasmid encoding a version of MARCH6 with a 3xFLAG tag at the NH_2_ terminus and a 1D4 tag at the COOH terminus (calculated molecular weight of 107 kDa). We then subjected lysates from these transfected cells to 8% SDS/PAGE and conducted immunoblot analysis using antibodies against the FLAG epitope, the 1D4 epitope, and MARCH6 (IgG-26F12c). As shown in Figure 6B, all three antibodies detected a band migrating between the 75 and 100 kDa molecular weight markers, suggesting that the NH_2_ and COOH termini of this overexpressed version of MARCH6 were intact. Based on these experiments, we conclude that the lower molecular mass of MARCH6 observed during 8% SDS/PAGE (the default acrylamide concentration used throughout this study) is likely due to anomalous migration during SDS-PAGE rather than posttranslational cleavage.

To further establish the specificity of IgG-26F12c in a cell line other than HuH7, we generated human fibroblast SV589 cells that were deficient in MARCH6. Using the same CRISPR-Cas9 genome editing strategy that produced HuH7 cells deficient in MARCH6 (Fig. 2A), we generated two SV589 cell lines that had disruptions in either exon 15 of MARCH6, designated as SV589 (MARCH6-KO1), or exon 4 of MARCH6, designated as SV589 (MARCH6-KO2) (Fig. 7A). Compared to SV589 (WT) cells, the two MARCH6 KO cell lines had increased levels of one of its substrates, SQLE, indicating that these two cell lines had diminished MARCH6 activity (Fig. 7B, *top panel*). Analysis with the MARCH6-specific IgG-26F12c antibody showed a single band in SV589 (WT) cells and this band was not detected in the two KO cell lines (Fig. 7B, *middle panel*, *compare lane 1 to lanes 2 and 3*). Thus, we have verified the specificity of IgG-26F12c in two different cell lines, HuH7 and SV589. Based on these results, we are confident that the single band observed in Sf9 insect cells, 3T3-L1 mouse cells, and A549, HEK293A, and HeLa human cells, and the doublet observed in CHO-K1 hamster cells (Fig. 5B) corresponds to endogenous MARCH6. We next conducted immunofluorescence analysis of SV589 cells with IgG-26F12C to examine the cellular localization of MARCH6. As shown in Figure 7C, staining by IgG-26F12C revealed that MARCH6 was localized to the ER of WT SV589 cells (*left column*) and this staining was markedly reduced in SV589 cells where MARCH6 was knocked out (*right column*). We also used IgG-26F12c to determine the distribution of March6 in various mouse tissues. As shown in Fig. 8, the liver contains the highest signal for March6 (*lane 1*), followed by the white and brown adipose tissue (*lanes 5, 6*). March6 is present at lower levels in other tissues and is conspicuously absent in muscle.

Finally, we expanded our characterization of IgG-26F12c to assess whether changes in cellular lipid composition affected levels of endogenous MARCH6. A previous study, using a human cell line (HEK293) modified to stably overexpress an epitope-tagged version of MARCH6, had suggested that increases in cellular cholesterol increased the levels of this epitope-tagged MARCH6 (19). Unfortunately, the authors of this study were unable to measure the levels of endogenous MARCH6 in a reliable manner due to the lack of a specific antibody. Now that we have generated a specific MARCH6 antibody (IgG-26F12c), we examined the stability of endogenous MARCH6 in five different human cell lines, including HEK293 cells. In the experiment shown in Figure 9, we used immunoblot analysis to compare cells that were grown in lipoprotein-rich serum (FCS) to cells that were grown in LPDS supplemented with compactin, an inhibitor of cholesterol synthesis. As expected, this method of cellular cholesterol depletion triggered the proteolytic cleavage of SREBP2 (*first panel*) and increased the levels of SQLE (*second panel*) (*compare lanes 1, 7, 13, 19, and 25 to lanes 2, 8, 14, 20, and 26, respectively*). Analysis of MARCH6 in all five cell lines under these conditions of cellular cholesterol excess and depletion showed no change in its levels (*third panel*, *compare lanes 1, 7, 13, 19, and 25 to lanes 2, 8, 14, 20, and 26, respectively*). When we replenished the cholesterol-depleted cells with cholesterol delivered in complexes with methyl-β-cyclodextrin (MCD) (30), we observed the expected termination of SREBP2 cleavage (*first panel*) and decrease in levels of SQLE in a dose-dependent manner (*second panel*) (*lanes 3–5, 9–11, 15–17, 21–23, and 27–29*). Replenishment with 25-hydroxycholesterol also terminated SREBP2 cleavage as expected (*first panel*, *lanes 6, 12, 18, 24, and 30*) and showed cell type–dependent variations in reduction of SQLE levels (*second panel*, *lanes 6, 12, 18, 24, and 30*). Once again, MARCH6 levels in all three cell lines were unchanged under these conditions of cholesterol or 25-hydroxycholesterol repletion (*third panel*). Thus, endogenous MARCH6 levels are unchanged in response to changes in cellular cholesterol content. The specific antibody developed in this study reveals how the behavior of endogenous MARCH6 is very different from that of epitope-tagged and overexpressed MARCH6.

## Discussion

MARCH6, an ER membrane–bound E3 ligase, has attracted much attention in the lipid research community as its substrates control varied lipid-related pathways including cholesterol biosynthesis and lipid droplet homeostasis (4, 5, 6, 7, 8, 9). However, studies of MARCH6 have been limited due to the lack of specific antibodies to monitor its levels in cells (Fig. 2B, C). The current studies overcome this limitation by generating a monoclonal antibody, IgG-26F12c, which specifically detects MARCH6 in cultured cells and mouse tissues (Fig. 4, Fig. 5, Fig. 7, Fig. 8). This advance was made possible by using full-length, purified MARCH6 (Fig. 3) as the immunogen, instead of peptides representing short portions of the protein that were used to generate the currently used antibodies that have poor specificity. This approach yielded IgG-26F12c, a specific antibody that detected a single band in WT, but not in MARCH6-KO cells (Fig. 4, Fig. 5, Fig. 7). The broad applicability of IgG-26F12c is indicated by its ability to detect MARCH6 in insect, mouse, hamster, and human cells (Fig. 5B), as well as in mice where it is present at highest levels in the liver (Fig. 8).

Following extensive characterization to establish the specificity of IgG-26F12c, we used it to address the question of whether levels of MARCH6 are sensitive to cellular cholesterol levels. In contrast to a previous report showing that epitope-tagged, overexpressed MARCH6 was stabilized by a rise in cellular cholesterol (19), we find that levels of endogenous MARCH6 (detected by IgG-26F12c) are unchanged in response to cellular cholesterol (Fig. 9). This result, which was made possible by our newly developed antibody, represents just one example of the detailed mechanistic studies of MARCH6 that can now be carried out and promises to provide many more insights into its cellular functions. Although we have focused on immunoblot analysis, it is possible that IgG-26F12c (or one of the other six purified antibodies shown in Fig. 5B) can also be used for immunofluorescence (the results of Fig. 7C are promising in this regard), immunoprecipitation, or immunohistochemistry studies, which would further broaden the scope of this reagent.

## Data availability

All data are presented within the article.

## Conflict of interest

The authors declare that they have no conflicts of interest with the contents of this article.
